# Effects of Different Light Sources on Neural Activity of the Paraventricular Nucleus in the Hypothalamus

**DOI:** 10.3390/medicina55110732

**Published:** 2019-11-09

**Authors:** Michio Yokoyama, Hyukki Chang, Hiroshi Anzai, Morimasa Kato

**Affiliations:** 1 Graduate School of Science and Engineering, Yamagata University, Yonezawa 992-8510, Japan; yoko@yz.yamagata-u.ac.jp; 2 Department of Human Movement Science, Seoul Women’s University, Seoul 01797, Korea; hkchang@swu.ac.kr; 3 Takahata Electronics Corporation, Yonezawa 992-0003, Japan; h-anzai@takahata-denshi.co.jp; 4 Department of Health and Nutrition, Yamagata Prefectural Yonezawa University of Nutrition Sciences, Yonezawa 992-0025, Japan

**Keywords:** organic light-emitting diode lighting, LED lighting, fluorescent lighting, color temperature, immunohistochemistry analysis, stress response

## Abstract

*Background and Objectives*: Physical function is influenced by light irradiation, and interest in the influence of light irradiation on health is high. Light signals are transmitted from the retina to the suprachiasmatic nucleus (SCN) via the retinal hypothalamic tract as non-image vision. Additionally, the SCN projects a nerve to the paraventricular nucleus (PVN) which acts as a stress center. This study examined the influences of three different light sources on neural activity in the PVN region using two different color temperatures. *Materials and Methods*: Experiments were conducted using twenty-eight Institute of Cancer Research (ICR) mice (10 week old males). Three light sources were used: (1) organic light-emitting diode (OLED) lighting, (2) LED lighting, and (3) fluorescent lighting. We examined the effects of light irradiation from the three light sources using two different color temperatures (2800 K and 4000 K). Perfusion was done 60 min after light irradiation, and then the brain was removed from the mouse for an immunohistochemistry analysis. c-Fos was immunohistochemically visualized as a marker of neural activity in the PVN region. *Results*: The number of c-Fos-positive cells was found to be significantly lower under OLED lighting and LED lighting conditions than under fluorescent lighting at a color temperature of 2800 K, and significantly lower under OLED lighting than LED lighting conditions at a color temperature of 4000 K. *Conclusions*: This study reveals that different light sources and color temperatures alter the neural activity of the PVN region. These results suggest that differences in the light source or color temperature may affect the stress response.

## 1. Introduction

Physical functions are regulated by lighting factors such as intensity, components of radiated light, and exposure duration. These light irradiations alter autonomic activity and the endocrine response, which control body temperature, the sleep–wake cycle, and also induce psychological changes [1,2,3,4].

Recently, more attention has been paid to the association between exposure to components of radiated light and health problems. In particular, the discovery of the photoreceptive intrinsically reactive retinal ganglion cells (ipRGCs) [5,6] has led to an increasing need for research on light irradiation and brain function. These cells are most sensitive to blue light [5], and neural projections from ipRGCs to the suprachiasmatic nucleus (SCN) through the retinohypothalamic tract have been observed [7,8,9,10,11]. Changes in the circadian rhythm and the inhibition of melatonin secretion caused by blue light are implicated in this pathway, because the SCN is a central circadian pacemaker [12,13,14]. The influence of blue light on health has been reported to improve cognitive function and reaction time [15,16,17]. Furthermore, blue light therapy has also been reported to be effective for the treatment of mild traumatic brain injury [18] and depression [19]. As described above, light irradiation may affect several regions in the brain. Indeed, neuronal projections in the brain are also projected from the SCN to the hypothalamic paraventricular nucleus (PVN), which is the center of stress regulation [20,21]. For this reason, light irradiation may also be related to stress responses.

In addition, color temperature (Kelvin), which is the scale used to measure emissions from a light source, is also an important factor in understanding the influence of light on physiological and psychological responses [22,23]. The color becomes blue when the temperature is higher and orange when the temperature is lower. Deguchi et al. [23] reported a contingent negative variation (CNV) response from electroencephalogram (EEG) of 11 human participants and found that a higher color temperature activated the reticular activating system more than a lower color temperature. Results from mice studies associated a higher color temperature with an increased stress and anxiogenic response [24]. These studies indicate that further research on light irradiation and brain function is needed to examine color temperature in more depth.

Considering our daily lives, the effects of light components and color temperature on brain function are mainly affected by differences in light sources such as fluorescent light, LED, and organic light-emitting diode (OLED). There is limited knowledge of the effects of different light sources on brain function. In a sleep study, Rahman et al. [25] compared fluorescent lighting with LED lighting when the blue light region was cut. They found that the power value of the delta-wave in the EEG signals was significantly elevated by blue-depleted LED lights compared with fluorescent lights. Additionally, Yuda et al. [26] found that relative to fluorescent lighting, OLED lighting was associated with lower heart rates and a greater high-frequency power component, which is an indicator of parasympathetic nerve activity. It was suggested that the changes in the psychological response and brain function were caused by the differences in the light source.

A non-image forming signal from the retina, which is received by the neural network and photoreceptors, is transmitted through the retinohypothalamic tract to the SCN [7,8,9,10,11], and neurons also project from the SCN to the PVN, which is the center of the stress regulation region. This study examined the effect of light irradiation produced by three different light sources on the neural activity of the hypothalamic/PVN area. We hypothesized that the difference in light source may alter the neural activity of the PVN.

## 2. Methods

### 2.1. Experimental Animals

Ten week old male Institute of Cancer Research (ICR) mice (*n* = 28) weighing between 32.3 and 47.1 g were obtained from CLEA, Japan, Inc. (Tokyo, Japan). All mice were housed singly in cages (125 mm × 113 mm × 197 mm) and maintained on a 12 h day/night cycle (light from 08:00 to 20:00 (a fluorescent lamp; 388 ± 13 lx) and darkness from 20:00 to 08:00) in an air-conditioned room with a temperature and humidity of 23 ± 2 degrees Celsius and 50% ± 10%, respectively. During the rearing period, the animals received commercial rodent chow (CE2, CLEA Japan Inc., Tokyo, Japan) and water ad libitum. For habituation to the laboratory conditions, the animals were housed for 2 weeks, and then the study was started. All procedures were performed in accordance with the Institutional Guidelines for Animal Care at the National Institute of Health Guidelines for the Care and Use of Laboratory Animals. The proposal for the use of animals was received and approved by the animal care review committee of the Yamagata Prefectural Yonezawa University of Nutrition Sciences (Approval Number 226, date of approval 30 October 2015).

### 2.2. Experimental Design

**Figure 1:**
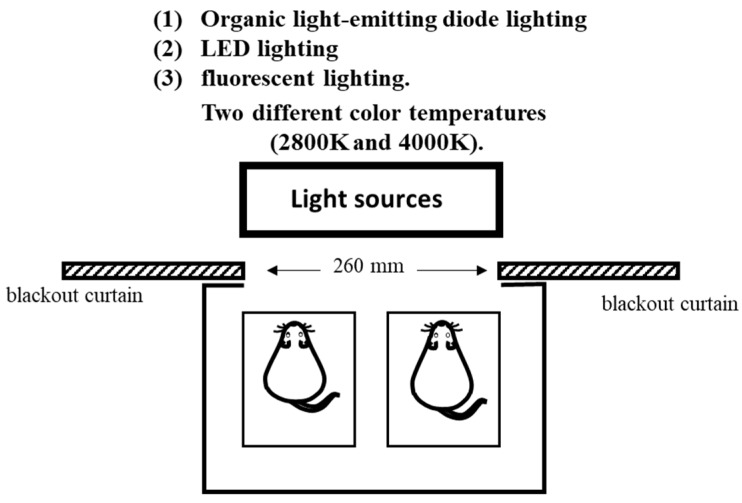
Light irradiation on mouse cage.

This study was conducted under light irradiation conditions using three different light sources and two different color temperatures. The following three light sources were used: (1) OLED lighting, (2) LED lighting, and (3) fluorescent lighting, and we examined the effects of light irradiation from the three light sources using two different color temperatures (2800 and 4000 K). The illumination intensity was set at 500 ± 10 lx for all light sources. Light irradiation was conducted under each condition for 1.5 h using each light source one hour before the start of the light period in order to minimize the effect on the circadian rhythm by eliminating the effect of the light history. During light irradiation, the light source was placed outside of the breeding equipment. A blackout curtain with an opening area of 75 mm × 260 mm was placed on the breeding equipment to maintain a constant level of light irradiation. When evaluating the spectral composition of the lighting source, the measurement position of the illuminance spectrophotometer and the cage position of the mouse during light irradiation were at the same distance. The mouse cage was transparent, and nothing in the breeding equipment absorbed light. Therefore, the light received by the mouse was similar to the state measured by the spectrophotometer (Figure 1).

### 2.3. Immunohistochemistry

Mice were anesthetized with isoflurane, perfused with 200 mL of 0.9% saline (at 60 min after the light emission), and then perfused with 300 mL of 4% paraformaldehyde in 0.1 M of phosphate buffered salts (PBS, pH 7.4). Then, the brains from the mice were quickly removed for immunohistochemistry analysis. The brain samples were placed in 20% sucrose, frozen on dry ice, and finally stored at −80 °C until sectioning. Frozen serial frontal sections (40 μm thick) were taken from the PVN. The brain sections were made using a cryo-microtome (CM1900; Leica Microsystems, Nussloch, Germany). An immunohistochemical visualization of c-Fos was carried out on free-floating sections using antibody and avidin-biotin-peroxidase methods. The free-floating sections were incubated with 0.3% H_2_O_2_, permeabilized with 0.3% Triton X-100, and nonspecific protein binding was blocked by incubation with 3% normal goat serum. The sections were incubated overnight at 4 °C with anti-c-Fos antibody (1:2000, rabbit polyclonal; Oncogene Research Products, San Diego, CA, USA). The sections were rinsed three times (10 min each) in phosphate-buffered saline with triton (PBT) and incubated with biotinylated goat anti-rabbit IgG (1:200; Vectastain Elite avidin-biotin complex (ABC) kit, Vector Laboratories, Burlingame, CA, USA) for 1 h. The sections were rinsed three times (10 min each) in PBT, incubated with ABC solution (1:50; Vectastain Elite ABC kit) for 1 h, and visualized using the diaminobenzidine (DAB) procedure method. The reaction was stopped by transferring the sections into 0.1 M acetate buffer and rinsing twice (5 min each) in PBT. After drying, the sections were mounted on glass slides using Eukitt (Kindler, Freiburg, Germany).

### 2.4. Data Analysis

#### 2.4.1. Light Source Analysis

The spectral compositions of the lighting sources were obtained for every lighting condition using an Illuminance Spectrophotometer (Konica Minolta CL-500A, Tokyo, Japan). The spectral power distribution, color temperature (Kelvin), and illuminance (lx) data were recorded at each cage position. The position of the illuminance spectrophotometer position was at the same distance as the cage position when the mouse was irradiated with light. In addition, quantification of the four photoreceptors’ (melanopsin, rods, and m- and s-cone) inputs in mice was conducted using the toolbox reported by Lucus’ group [27,28].

#### 2.4.2. Analysis of the c-Fos-Positive Cell Number

The tissue sections were scanned using an All-in-One Fluorescence Microscope (BZ-X700, Keyence, Osaka, Japan). The images obtained from the PVN in a mouse brain atlas were overlaid using paint.net, which is an image and photo editing software. Then, we manually counted the number of c-Fos positive cells in the PVN.

### 2.5. Statistics

We calculated the mean and standard deviation (SD) of the number of brain cells by light source and by color temperature. Then, we analyzed the primary effects of the light source and color temperature and their interactions by two-way ANOVA using the number of brain cells as the dependent variable and the light source, color temperature, and the interaction between light source and color temperature as independent variables. Subsequently, we performed a multiple comparisons test using Tukey’s honestly significant difference (HSD) method to compare the light sources based on the results of the variance analysis, and we performed an unpaired t-test to compare the color temperatures. A statistical analysis was performed using the SPSS statistics package software, version 22. (IBM Japan, Tokyo, Japan). A risk rate of less than 5% was regarded as representing a significant difference.

## 3. Results

### 3.1. Light Irradiation

**Figure 2:**
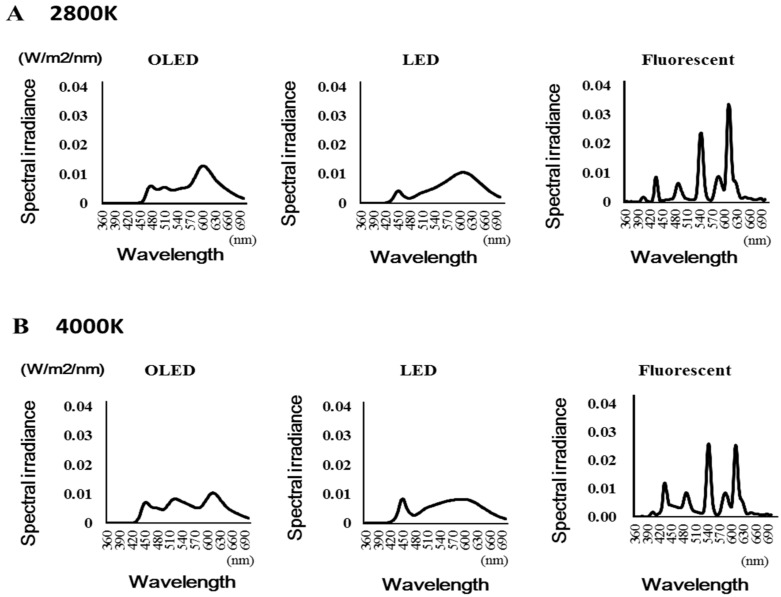
Spectral composition of each light source. (**A**): 2800 Kelvin (**B**): 4000 Kelvin.

Figure 2 shows the spectral composition of each light source. The peak wavelength of each light source was as follows: The peak wavelength of the OLED light was 601.7 nm at 2800 K (602, 601, and 602 nm for the three measurements) and 613.5 nm at 4000 K (613 and 614 nm for the two measurements). The peak wavelength of the LED light was 609 nm at 2800 K (609 and 609 nm for the two measurements) and 450 nm at 4000 K (450 and 450 nm for the two measurements). The peak wavelength of the fluorescent light was 612 nm at 2800 K (612 and 612 nm for the two measurements) and 544 nm at 4000 K (544 and 544 nm for the two measurements).

**Figure 3:**
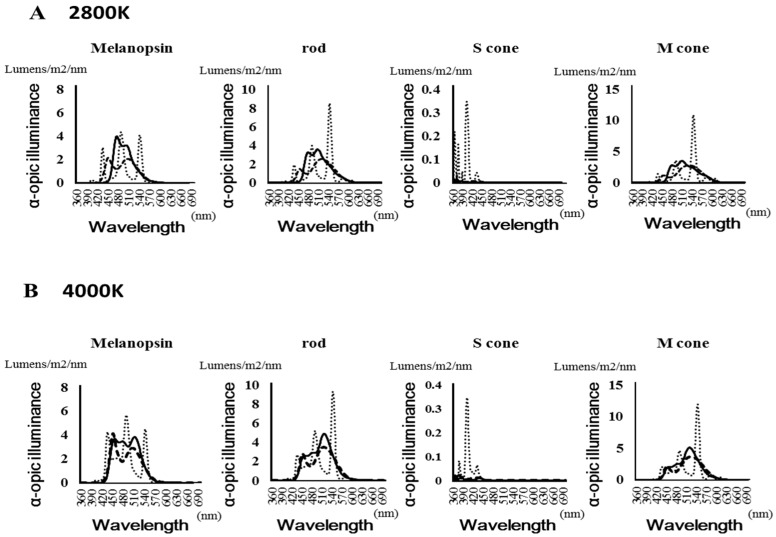
Spectral distribution of the α-opic lux values of each photoreceptor. (**A**): 2800 Kelvin (**B**): 4000 Kelvin.

Figure 3 shows the spectral distribution of the α-opic lux values of each photoreceptor (melanopsin, rods, and m- and s-cones). Table 1 shows the maximum value of the α-opic lux of each photoreceptor and the frequency. The LED had a peak at around 450 nm when evaluated with the melanopsin receptor. Under fluorescent light, the S-cone showed a value more than 10 times that of the other light sources.

The solid line is OLED, the thick dotted line is LED light, and the fine-dotted line is fluorescent light.

### 3.2. c-Fos-Positive Cell Number

**Figure 4:**
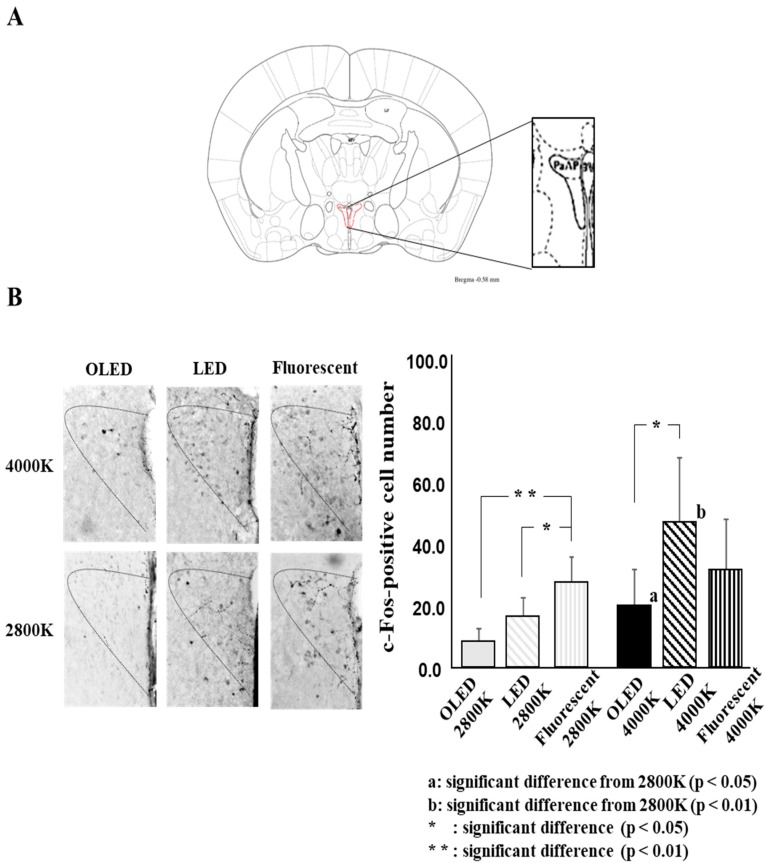
Comparison of each light source, comparison of color temperature. (**A**) Analysis of brain region (**B**) c-Fos-positive cells number for each light source at two color temperature. All data on the figure expressed as mean ± SD. a: significant difference from 2800 K (*p* < 0.05), b: significant difference from 2800 K (*p* < 0.01), *: significant difference (*p* < 0.05), **: significant difference (*p* < 0.01).

Figure 4 shows the c-Fos-positive cell number for each light source, as follows: For OLED light, 8.4 ± 4.3 at 2800 K and 20.0 ± 11.8 at 4000 K; for LED light, 16.6 ± 5.9 at 2800 K and 47.1 ± 20.8 at 4000 K; and for fluorescent light, 27.4 ± 8.4 at 2800 K and 31.3 ± 16.9 at 4000 K.

The two-way ANOVA gave the following results: light source: F ((2.36) = 5.8, *p* = 0.006), color temperature: F((1, 36) = 12.5, *p* = 0.001), and light source × color temperature: F ((2, 36) = 3.7, *p* = 0.034)).

The comparison of the light sources revealed a significant difference between OLED lighting and fluorescent lighting and between LED lighting and fluorescent lighting for the 2800 K lamp and between OLED lighting and LED lighting for the 4000 K lamp. The comparison of color temperature showed a significant difference between OLED lighting and LED lighting, with the 4000 K lamp showing significantly higher positive numbers than the 2800 K lamp.

## 4. Discussion

Using two different color temperatures, this study revealed, for the first time, that a difference in light source can alter neural activity in the stress-related region of the hypothalamic area. It was found that the c-Fos expression within PVN at each color temperature was lowest under OLED light conditions compared with the other tested light source conditions. Furthermore, an increase in color temperature led to a significant increase in c-Fos expression within PVN in OLED and LED conditions. The above results suggest that differences in the light source or color temperature may affect the stress response.

In this study, we confirmed that c-Fos expression was lower in the PVN under OLED light compared with other light sources when the color temperature was the same for each light source. It is speculated that this factor may have be caused by the effect of blue light on the ipRGCs and s-cone photoreceptors. Since it is considered that light from an external source is received by photoreceptors, it becomes a neural signal which is transmitted through the retinohypothalamic tract to the SCN [7,8,9,10,11]. Furthermore, this signal is transmitted from the SCN to the PVN, which controls the physiological functions through endocrine and autonomic responses [19,20,29]. A previous study reported a more efficient response of the ipRGCs to blue light compared to other photoreceptors, and it is believed that the wavelength region of 440–450 nm has the highest level of blue light energy [30]. Mei-Ling Peng et al. [31] examined the impacts of domestic LED lamps on the retinas of mice and found that peak retinal degeneration occurred at 450 nm within the wavelength range of blue light. Moreover, this study indicated that the melanopsin-integrated α-opic lux value of 440–450 nm was highest under the LED condition and lowest under OLED. Therefore, it is speculated that the difference in irradiance in this wavelength range may affect c-Fos expression in the PVN, and this difference may explain the low expression level of c-Fos under the OLED condition.

In addition, it is suggested that the color temperature may affect the expression of c-Fos in the PVN. Previous human studies reported enhancement of sympathetic nerve activity and the arousal level and suppression of melatonin secretion by increasing the color temperature [32]. Animal studies also reported an association between higher frequencies of stress behaviors and an increase in color temperature [24]. This study linked an increase in color temperature to higher c-Fos expression for each light source. Particularly in LED and OLED conditions, a significant increase in c-Fos expression was found due to an increase in color temperature. However, in this study, we only found a small increase in c-Fos expression under fluorescent conditions following the increase in the color temperature. This factor may be responsible for the effect in the UV region from the fluorescent lamp light source. In addition, the results of this study indicate that wavelengths lower than 400 nm are associated with high spectral irradiance under fluorescent conditions compared with other light conditions. Previous reports have indicated that mice have ultraviolet cone receptors and are highly sensitive to short-wavelength light [33]. In fact, the s-cone α-opic lux value in this study showed a more than 10-fold increase under fluorescent lamp conditions compared with the other conditions. Only a small increase in c-Fos expression was reported following the increase in color temperature, because c-Fos expression increased even at a low color temperature under fluorescent conditions compared with the other light conditions.

Stress-related cardiovascular responses, endocrine responses, and autonomic responses are controlled by the PVN, the target of this study [34,35,36]. Previous studies in humans have reported that different light sources or color temperatures may alter the autonomic activity, psychological state, and working performance [22,23,24,25,26]. For example, it has been indicated that sympathetic nerve activity is increased at high color temperatures in association with autonomic activities [22]. In addition, it has been reported that parasympathetic nerve activity is enhanced to a greater extent under OLED light than under fluorescent light [26]. In this study, marked differences in c-Fos expression were observed in the PVN with different light sources. This suggests that the light source may affect the stress response, and this may be lower under OLED light.

This study has several limitations. First, it was not intended to evaluate the damage on the retina caused by light stimuli but rather, was intended to evaluate neural activity in the PVN, which plays a key role in the stress response. Therefore, the irradiation time was short in this study, compared with studies where damage was caused to the retina [37,38,39,40,41]. This may have occurred in response to weak light stimuli in mice. Further studies need to be carried out to assess whether there is a difference between light sources on the level of light stimulation, which can cause damage to the retina. Secondly, we did not measure physiological and/or behavioral data for light stimuli. Subsequent studies need to be carried out to assess the physiological response to light in order to examine the detailed association between the difference in the stress reaction following exposure to various light sources. Furthermore, in order to examine the physiological mechanism, it is also necessary to consider factors including those related to each photoreceptor. In this study, we observed changes in neural activity in the PVN that were associated with different light sources. However, it is unknown which neural cells are involved in the release of hormones from in the PVN, including vasopressin, corticotropin-releasing hormone (CRH), and oxytocin. Thus, there are some limitations in this study. Future research studies should address these limitations.

## 5. Conclusions

In this study, the experiment was examined the influences of three different light sources on neural activity in the PVN region using two different color temperatures. This study carried out with the same illumination intensity under all light source conditions. Therefore, the differences in c-Fos expression in the PVN are presumed to have been due to the difference in the spectral composition of the light sources. Light irradiation from each light source had a characteristic effect on each photoreceptor. The effect on the s-cone was strong with fluorescent lamp irradiation and the effect on melanopsin was strong under the LED condition and weak under the OLED condition at 440–450 nm, which is the blue wavelength energy region. The hypothalamic PVN region is involved in stress regulation, and the results of this study suggest that differences in light source and color temperature affect the stress response.

## Figures and Tables

**Table 1:** Maximum value of the α-opic lux and frequency of each photoreceptor.

		Melanopsin		Rods		S cone		M cone	
Color Temperature	Light Source	α-Opic Lux Value	Peak Wavelength	α-Opic Lux Value	Peak Wavelength	α-Opic Lux Value	Peak Wavelength	α-Opic Lux Value	Peak Wavelength
		(Lumens/m^2^/nm)	(nm)	(Lumens/m^2^/nm)	(nm)	(Lumens/m^2^/nm)	(nm)	(Lumens/m^2^/nm)	(nm)
2800 K	OLED	3.973	477	3.552	507	0.023	360	3.47	509
	LED	2.143	452	2.513	519	0.029	372	2.75	531
	Fluorescent	4.362	489	8.506	543	0.347	402	10.888	544
4000 K	OLED	3.854	512	4.887	517	0.015	387	5.11	520
	LED	4.11	452	3.477	516	0.03	368	3.672	525
	Fluorescent	5.719	489	9.345	543	0.348	402	11.913	543

(O)LED: (organic) light emitting diode.
